# 3D analysis of apparent diffusion coefficient histograms in hepatocellular carcinoma: correlation with histological grade

**DOI:** 10.1186/s40644-016-0103-3

**Published:** 2017-01-05

**Authors:** Tomohisa Moriya, Kazuhiro Saito, Yu Tajima, Taiyo L. Harada, Yoichi Araki, Katsutoshi Sugimoto, Koichi Tokuuye

**Affiliations:** 1 Department of Radiology, Tokyo Medical University, 6-7-1 Nishishinjuku, Shinjuku-ku, Tokyo, 160-0023 Japan; 2 Department of Gastroenterology and Hepatology, Tokyo Medical University, Tokyo, Japan

**Keywords:** Hepatocellular carcinoma, Diffusion-weighted imaging, ADC histogram, Histological differentiation

## Abstract

**Background:**

To evaluate the usefulness of differentiation of histological grade in hepatocellular carcinoma (HCC) using three-dimensional (3D) analysis of apparent diffusion coefficient (ADC) histograms retrospectively.

**Methods:**

The subjects consisted of 53 patients with 56 HCCs. The subjects included 12 well-differentiated, 35 moderately differentiated, and nine poorly differentiated HCCs. Diffusion-weighted imaging (*b*-values of 100 and 800 s/mm^2^) were obtained within 3 months before surgery. Regions of interest (ROIs) covered the entire tumor. The data acquired from each slice were summated to derive voxel-by-voxel ADCs for the entire tumor. The following parameters were derived from the ADC histogram: mean, standard deviation, minimum, maximum, mode, percentiles (5th, 10th, 25th, 50th, 75th, and 90th), skew, and kurtosis. These parameters were analyzed according to histological grade. After eliminating steatosis lesions, these parameters were re-analyzed.

**Results:**

A weak correlation was observed in minimum ADC and 5th percentile for each histological grade (*r* = –0.340 and *r* = –0.268, respectively). The minimum ADCs of well, moderately, and poorly differentiated HCC were 585 ± 388, 411 ± 278, and 235 ± 102 × 10^−6^ mm^2^/s, respectively. Minimum ADC showed significant differences among tumor histological grades (*P* = 0.009). The minimum ADC of poorly differentiated HCC and that of combined well and moderately differentiated HCC were 236 ± 102 and 437 ± 299 × 10^−6^ mm^2^/s. The minimum ADC of poorly differentiated HCC was significantly lower than that of combined well and moderately differentiated HCC (*P* = 0.001). The sensitivity and specificity, when a minimum ADC of 400 × 10^−6^ mm^2^/s or lower was considered to be poorly differentiated HCC, were 100 and 54%, respectively. After exclusion of the effect of steatosis, the sensitivity and specificity did not change, although the statistical differences became strong (*P* < 0.0001).

**Conclusion:**

Minimum ADC was most useful to differentiate poorly differentiated HCC in 3D analysis of ADC histograms.

## Background

The histological grade of hepatocellular carcinoma (HCC) is a major contributing factor to recurrence after surgery, and poorly differentiated HCC tends to have higher recurrence rates than well and moderately differentiated HCC [1, 2]. Therefore, distinguishing the histological grade of HCC before therapy can be effective to create a therapeutic strategy and estimate the prognosis. It has been previously reported that poorly differentiated HCC showed decreasing arterial blood flow using CT hepatic arteriography [3]. However, the decreased arterial blood supply is observed in not only poorly differentiated HCC but also in well differentiated HCC [4], and this makes the discrimination of these two entities difficult. To clearly distinguish tumor histological grade, diffusion-weighted imaging (DWI) that is independent of vascularity has been proposed. Some papers have already reported about the diagnosis of tumor histological grade using the apparent diffusion coefficient (ADC). However, the methodology and results were variable and inconsistent [5–9]. The region of interest (ROI) setting is significant to eliminate bias, for example, if the ROI is set in the tumor avoiding areas of necrosis, this measurement involves arbitrariness of the researchers. Furthermore, when the ROI is set at the entire tumor on a slice, the measured ADC is represented only on the selected slice. Some different histological grade components are often included in an HCC nodule; therefore, the ROI set through the entire lesion three-dimensionally may lead to a more accurate diagnosis. The usefulness of differentiation of brain glioma grade using ADC histogram analysis in which ROI is set to the entire lesion three-dimensionally has already been reported [10]. We evaluated the usefulness of differentiating the histological grade of HCC using 3D analysis of ADC histograms derived from the ROI set at the entire tumor.

## Methods

This retrospective study was approved by an institutional review board and informed consent was waived.

### Subjects

The researchers referred to medical records and a radiological database and the eligibility criteria were determined as follows: (a) the same parameter of DWI, (b) resected and pathologically confirmed HCC, (c) patients previously not receiving radiofrequency ablation and trans-arterial chemoembolization, (d) MRI was performed within 3 months prior to surgery. Exclusion criteria were as follows: (a) patients with artifacts associated with body metal and/or body movement, (b) patients whose tumors are present at the left lobe lateral segment, (c) boundary of the tumor is unclear due to an infiltrative feature. Finally, 52 patients with 56 nodules were enrolled in this study, which included 41 men and 11 women with a mean age of 68 years and a median age of 66 years. Underlying liver diseases were hepatitis B (*n* = 6), hepatitis C (*n* = 13), non-B non-C hepatitis (*n* = 1), alcoholic liver (*n* = 12), non-alcoholic steatohepatitis (*n* = 1), fatty liver (*n* = 1), primary sclerosing cholangitis (*n* = 2), idiopathic portal hypertension (*n* = 1), and absence of underling liver disease (*n* = 15). Partial hepatectomy, liver sub-segmentectomy, segmentectomy, and lobectomy were performed in 30, 7, 11, and 4 patients, respectively. All patients were classified into Child-Pugh “A”. Makuuchi criteria were used to select the surgical indication [11]. The indocyanine green test (ICG15) and technetium-99 m diethylenetriamine pentaacetic acid galactosyl human serum albumin single photon emission computed tomography were also performed to evaluate liver functional reserve. Four patients had two tumors in a resected liver. Histological grade of HCC was classified into well, moderately, and poorly differentiated HCC [12]. When multiple components of histological grade were contained within a lesion, the major component was regarded as the tumor grade. Finally, this study included 12 well, 35 moderately, and nine poorly differentiated HCCs.

### MRI protocols

All magnetic resonance imaging examinations were performed with a 1.5 T superconductive MRI system (Avanto, Siemens, Erlangen, Germany) with a 32-channel body phased-array coil. The maximum gradient strength of the system was 45 mT/m and the maximum slew rate was 200 T/m/s. The non-contrast T1-weighted imaging (T1WI) was performed using a breath-hold two-dimensional gradient-echo sequence with the following parameters: repetition time/echo time, (TR/TE), 125 ms/2.38 ms for opposed phase and 4.76 ms for in-phase; flip angle, 75°; slice thickness, 5 mm; intersection gap, 1 mm; matrix, 320 × 154; field of view (FOV), 400 mm × 446 mm; average, 1; bandwidth, 470 Hz/pixel. The T2-weighted imaging (T2WI) was performed using the navigator-assisted technique for respiratory gating (2D-PACE). The T2WI parameters were as follows: TR/TE, 1600 ms/81 ms; flip angle, 150°; matrix, 512 × 176; FOV, 400 mm × 447 mm; slice thickness, 5 mm; intersection gap, 1 mm; average, 1; bandwidth, 260 Hz/pixel; fat suppression, chemical shift selective (CHESS). DWI was performed using a spin-echo-based echo-planar imaging sequence. The parameters were as follows: TR/TE, 1600 ms/66 ms; *b*-values, 100 and 800 s/mm^2^; matrix, 128 × 124; FOV, 400 mm × 447 mm; slice thickness, 5 mm; intersection gap, 1 mm; average, 4; bandwidth, 260 Hz/pixel; fat suppression, CHESS; using the navigator-assisted technique for respiratory gating (2D-PACE). For dynamic and hepatobiliary MRI, gadolinium-ethoxybenzyl-diethylenetriamine pentaacetic acid (Gd-EOB-DTPA) (Primovist, Bayer Schering; 0.025 mmol/kg) was rapidly administered intravenously and immediately followed by 20 mL of sterile saline flush with an injector at 1.0–2.0 mL/s. The hepatobiliary phase images were acquired at 20 min after contrast media injection by three-dimensional volumetric interpolated breath-hold examination (3D-VIBE). The sequence parameters were the following: TR/TE, 3.3 ms/1.2 ms; flip angle, 15.0°; matrix, 320 × 165; FOV, 400 mm × 446 mm; slice thickness, 2–3 mm; average, 1; intersection gap, 0 mm; fat suppression, CHESS.

### Image analysis

**Fig. 1:**
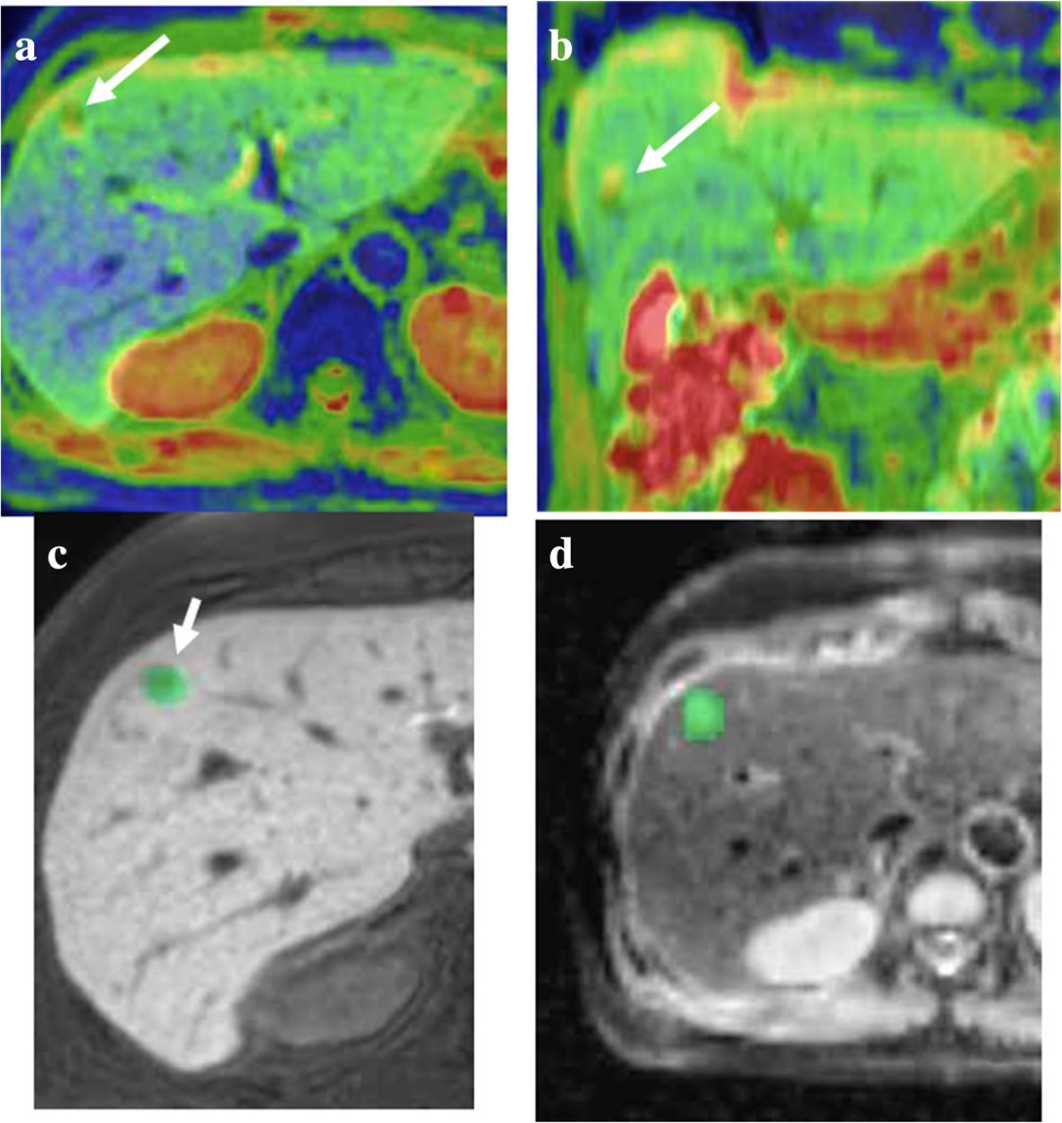
Process of making the ADC histogram. **a**, **b** To match the location on the hepatobiliary phase with that on the ADC map, the hepatobiliary phase is superimposed on the ADC map automatically. However, this fusion imaging has some gap and, therefore, some manual correction is necessary. **c**, **d** The ROIs were set at the entire tumor through all slices on hepatobiliary phase images (**c**). Then, the information of this ROI setting was copied and pasted on ADC maps (**d**)

**Fig. 2:**
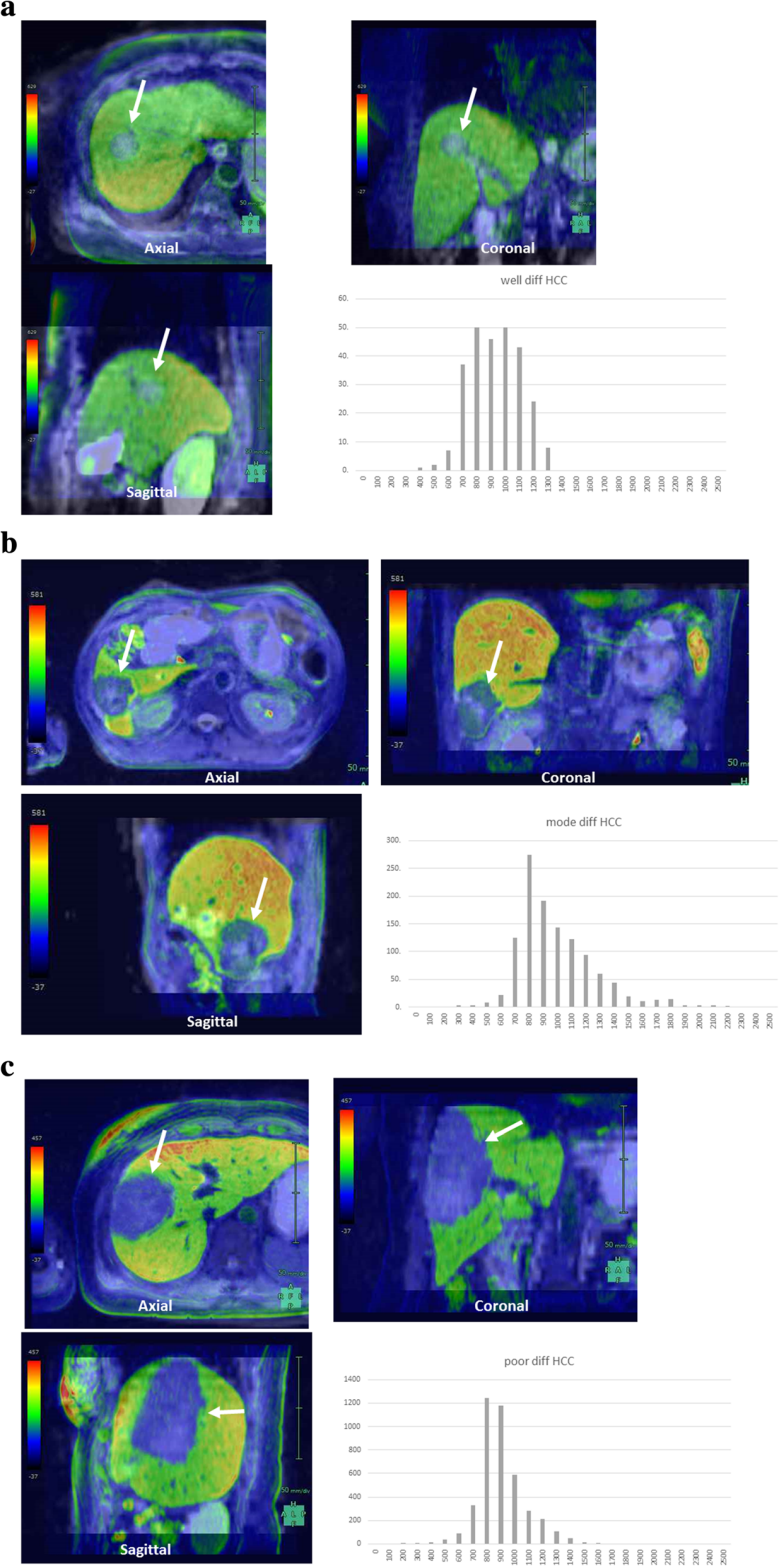
The representative cases of ADC histogram and 3D ADC map in (**a**) well, (**b**) moderately, and (**c**) poorly differentiated HCC are shown. Smaller minimum ADCs are present in poorly differentiated HCC compared with the other two histological grades. The data acquired from each slice were summated to derive voxel-by-voxel ADC values for the entire tumor and the ADC histogram was generated. Arrows indicates tumor

Two radiologists (with 4 and 25 years of experience) identified HCC on both images at the hepatobiliary phase and T2WI. Boundary of HCC was defined as a range of low signal intensity at the hepatobiliary phase. In case of revealing both high and low intensity in a lesion at the hepatobiliary phase, the region showing a high intensity in T2WI was defined as HCC. First, the radiologist (4 years of experience) and the radiological technologist (20 years of experience) attempted to match the location on the hepatobiliary phase (or T2WI) with that on the ADC map. They delineated the ROI on hepatobiliary phase or T2-weighted images, because the contour of the tumor was usually blurred on DWI. The hepatobiliary phase (or T2-weighted image) is superimposed on the ADC map automatically at the workstation (Synapse Vincent). However, this fusion imaging has some gap and, therefore, some manual correction is necessary (Fig. 1a, b). Second, the ROIs were set at the entire tumor through all slices on hepatobiliary phase images (Fig. 1c). Third, the information of the ROI setting on the hepatobiliary phase was copied and pasted on the ADC maps (Fig. 1d). Finally, the data acquired from each slice were summated to derive voxel-by-voxel ADC values for the entire tumor and an ADC histogram was generated (Fig. 2). Since the impact of steatosis on ADC has been reported previously, two radiologists (with 4 and 25 years of experience) established a consensus reading and the concomitant fat deposit was classified into the following three categories by chemical shift imaging: “1”, absence of signal decrease; “2”, signal decrease in less than half of the tumor; “3”, distinct decrease of the signal on more than half of the tumor.

### Statistical analysis

The mean, standard deviation, minimum, maximum, mode, skewness, kurtosis, and percentiles (5th, 50th, 75th, and 90th) were derived from the ADC histogram. Correlation between the degree of tumor histological grade and the parameters of ADC histograms were assessed by the Pearson’s product moment correlation coefficient. The differences in parameters among tumor histological grades were analyzed by one-way ANOVA with the Tukey-Kramer post hoc test. Poorly differentiated HCC and the group combining well and moderately differentiated HCC were compared using an unpaired Student *t*-test. In addition, in order to eliminate the interference from steatosis as much as possible, the parameters of the ADC histograms were re-analyzed in the same way as above after excluding the classified cases into “3” on the evaluation of chemical shift imaging. A *P* value < 0.05 was considered to be significant. ROC curves were created for the parameters found to be significant from these results, and then the sensitivity and the specificity were calculated by the Youden index. All statistical analyses were performed using SPSS statistics software (version 22, SPSS) for Microsoft Windows.

## Results

### Overall analysis

Table 1 shows the summary of parameters of ADC histograms associated with each histological grade. Weak correlations were found in minimum ADC and 5th percentile ADC (*r* = –0.340 and *r* = –0.268, respectively).

**Table 1:** Histological grade of hepatocellular carcinoma, the parameters of ADC histograms, Pearson’s product moment correlation coefficient, and one-way ANOVA with the Tukey-Kramer post hoc test

	Well diff HCC	Mod diff HCC	Poor diff HCC	Pearson’s correlation coefficient	Pearson’s correlation coefficient *P* value	ANOVA Tukey-Kramer post hoc test
Mean	1051 ± 203	999 ± 163	964 ± 167	−0.141	0.301	n.s.
Standard deviation	180 ± 119	193 ± 86	227 ± 75	0.154	0.257	n.s.
Minimum	585 ± 388	411 ± 278	235 ± 102	−0.340	0.010*	0.009**
Maximum	1641 ± 558	1718 ± 571	1955 ± 704	0.156	0.252	n.s.
Mode	710 ± 517	881 ± 311	829 ± 319	0.074	0.588	n.s.
Skewness	0.58 ± 1.27	0.36 ± 0.88	0.50 ± 1.00	−0.011	0.936	n.s.
Kurtosis	4.75 ± 4.59	4.86 ± 3.14	5.37 ± 3.23	0.056	0.681	n.s.
5th percentile	822 ± 216	718 ± 178	642 ± 147	−0.268	0.046*	n.s.
50th percentile	1034 ± 198	992 ± 160	936 ± 154	−0.170	0.211	n.s.
75th percentile	1149 ± 258	1112 ± 181	1078 ± 187	−0.104	0.448	n.s.
90th percentile	1248 ± 293	1234 ± 223	1244 ± 244	−0.002	0.990	n.s.

Well diff HCC: Well differentiated hepatocellular carcinoma

Mod diff HCC: Moderately differentiated hepatocellular carcinoma

Poor diff HCC: Poorly differentiated hepatocellular carcinoma

*, ** *P* < 0.05

n.s. no significant difference

**Tukey-Kramer post hoc test: Well diff HCC vs Mod diff HCC, *P* = 0.521; Well diff HCC vs Poor diff HCC, *P* = 0.121; Mod diff HCC vs Poor diff HCC, *P* = 0.008

Minimum ADC showed significant differences among tumor histological grades (*P* = 0.009). The other parameters did not show significant differences. The post hoc test showed significant differences between moderately and poorly differentiated HCC (*P* = 0.008) (Table 1).

The parameters of ADC histograms of poorly differentiated HCC and well and moderately differentiated HCC are shown in Table 2. Minimum ADC showed a significant difference (*P* = 0.001). The sensitivity and specificity, when a minimum ADC of 400 × 10^−6^ mm^2^/s or lower was considered to be poorly differentiated HCC, were 100 and 54%, respectively.

**Table 2:** Comparison of well differentiated and moderately differentiated hepatocellular carcinoma with poorly differentiated hepatocellular carcinoma using the parameters of ADC histograms

	Well + Mod diff HCC	Poor diff HCC	*P* value
Mean	1007 ± 168	964 ± 167	0.470
Standard deviation	191 ± 90	227 ± 75	0.251
Minimum	437 ± 299	235 ± 102	0.001*
Maximum	1706 ± 563	1955 ± 704	0.232
Mode	855 ± 348	829 ± 319	0.829
Skewness	0.40 ± 0.94	0.50 ± 1.00	0.752
Kurtosis	4.84 ± 3.34	5.37 ± 3.23	0.648
5th percentile	733 ± 186	642 ± 147	0.151
50th percentile	998 ± 164	936 ± 154	0.276
75th percentile	1118 ± 192	1078 ± 187	0.552
90th percentile	1236 ± 231	1244 ± 244	0.924

Well + Mod diff HCC: Well differentiated and moderately differentiated hepatocellular carcinoma

Poor diff HCC: Poorly differentiated hepatocellular carcinoma

*P < 0.05

### Excluded steatosis lesion

The signal intensity reduction on chemical shift imaging was classified as follows: 1, 41 nodules; 2, 4 nodules; 3, 11 nodules. Of the 11 classified into grade 3, 2 well, 8 moderately, and 1 poorly differentiated HCC were histologically included. The summaries of parameters of ADC histograms, except for grade 3 nodules, are shown in Table 3. Weak correlations were found in minimum ADC and 5th percentile ADC (*r* = –0.469 and *r* = –0.382, respectively).

**Table 3:** Histological grade of hepatocellular carcinoma (after excluding steatosis lesions), the ADC histogram parameters, Pearson’s product moment correlation coefficient, and one-way ANOVA with the Tukey-Kramer post hoc test

	Well diff HCC	Mod diff HCC	Poor diff HCC	Pearson’s correlation coefficient	Pearson’s correlation coefficient *P* value	ANOVA Tukey-Kramer post hoc test
Mean	1071 ± 178	1004 ± 157	967 ± 176	−0.164	0.281	n.s.
Standard deviation	147 ± 86	196 ± 89	243 ± 76	0.290	0.053	n.s.
Minimum	738 ± 322	421 ± 271	221 ± 110	−0.469	0.001*	0.006**
Maximum	1588 ± 493	1787 ± 579	1808 ± 606	0.201	0.186	n.s.
Mode	582 ± 531	890 ± 281	799 ± 347	0.122	0.425	n.s.
Skewness	0.91 ± 1.40	0.54 ± 0.84	0.59 ± 0.94	−0.073	0.633	n.s.
Kurtosis	5.39 ± 5.45	5.10 ± 3.38	5.50 ± 3.61	0.017	0.912	n.s.
5th percentile	910 ± 187	734 ± 181	631 ± 158	−0.382	0.010*	0.03***
50th percentile	1042 ± 163	997 ± 154	934 ± 160	−0.192	0.205	n.s.
75th percentile	1139 ± 222	1119 ± 173	1089 ± 199	−0.078	0.610	n.s.
90th percentile	1218 ± 217	1244 ± 219	1271 ± 260	0.065	0.672	n.s.

Well diff HCC: Well differentiated hepatocellular carcinoma

Mod diff HCC: Moderately differentiated hepatocellular carcinoma

Poor diff HCC: Poorly differentiated hepatocellular carcinoma

*, **, *** *P* < 0.05

n.s. no significant difference

**Tukey-Kramer post hoc test: Well diff HCC vs Mod diff HCC, *P* = 0.187; Well diff HCC vs Poor diff HCC, *P* = 0.045; Mod diff HCC vs Poor diff HCC, *P* = 0.008

***Tukey-Kramer post hoc test: Well diff HCC vs Mod diff HCC, *P* = 0.110; Well diff HCC vs Poor diff HCC, *P* = 0.023; Mod diff HCC vs Poor diff HCC, *P* = 0.317

Minimum ADC and 5th percentile ADC showed significant differences among three histological grades (*P* = 0.006 and 0.030, respectively). The other parameters did not show significant differences. The post hoc test of minimum ADC showed significant differences between well and poorly differentiated, and between moderately and poorly differentiated HCC (*P* = 0.045 and 0.008, respectively). The 5th percentile ADC of poorly differentiated HCC was significantly lower than that of well differentiated HCC (*P* = 0.023) (Table 3).

Minimum ADC showed significant differences between poorly differentiated HCC and well and moderately differentiated HCC (*P* < 0.0001) (Table 4). The sensitivity and specificity, when a minimum ADC of 400 × 10^−6^ mm^2^/s or lower was considered to be poorly differentiated HCC, were 100 and 54%, respectively.

**Table 4:** Comparison of well differentiated and moderately differentiated hepatocellular carcinoma with poorly differentiated hepatocellular carcinoma using the parameters of ADC histograms (after excluding steatosis lesions)

	Well + Moddiff HCC	Poor diff HCC	*P* value
Mean	1013 ± 159	967 ± 176	0.474
Standard deviation	189 ± 89	243 ± 76	0.123
Minimum	464 ± 295	221 ± 110	<0.0001*
Maximum	1760 ± 566	2031 ± 769	0.256
Mode	849 ± 333	799 ± 347	0.704
Skewness	0.59 ± 0.92	0.59 ± 1.11	0.999
Kurtosis	5.14 ± 3.63	5.50 ± 3.61	0.799
5th percentile	758 ± 189	631 ± 158	0.085
50th percentile	1003 ± 154	934 ± 160	0.258
75th percentile	1122 ± 177	1089 ± 199	0.640
90th percentile	1241 ± 216	1271 ± 260	0.728

Well + Mod diff HCC: Well differentiated and moderately differentiated hepatocellular carcinoma

Poor diff HCC: Poorly differentiated hepatocellular carcinoma

*P < 0.05

## Discussion

Minimum ADC was useful to distinguish poorly differentiated HCC from well and moderately differentiated HCC because minimum ADC and 5th percentile ADC were weakly correlated with histological grades; furthermore, minimum ADC showed significant differences among tumor histological grades. Nakanishi et al also reported that minimum ADCs in poorly differentiated HCC were significantly lower than those in the other histological grades, although they did not perform ADC histogram analysis [2]. Minimum ADC may reflect the hypercellular component in the tumor. As tumor histological grade increases, the cellularity of the tumor usually increases. This leads to restricted diffusion. We suppose that the results of the present study support this assumption.

Minimum ADC provided good sensitivity of 100%; however, there was a low specificity of 54% on diagnosis of poorly differentiated HCC. Although several researchers had previously reported the histological grade of the tumor using DWI, the accuracy was relative low. Nishie et al conducted the diagnosis of poorly differentiated HCC using mean ADC and obtained sensitivity of 73.1% and specificity of 72.9% [8]. The ROIs were put on the lowest intensity area in the solid part in the tumor on the ADC map in their report. In contrast, the ROI were set at entire tumors in our examination; therefore, we believe our results are more reproducible. We suppose that the low specificity in the present study was due to the variety of histological structure in well differentiated HCC. Minimum ADC showed significant differences between moderately differentiated HCC and poorly differentiated HCC; however, there were no differences between well differentiated HCC and moderately or poorly differentiated HCC. One of the reasons for this result might be that the minimum ADC of well differentiated HCC had wide variation (actually its standard deviation was large and the skewness was higher, although there was no significant difference). Moreover, the low mode ADC in well differentiated HCC indicates a large amount of histological components that showed restricted diffusion. Well differentiated HCC covers a variable range, from early HCC that is histologically similar to surrounding non-tumorous hepatic tissue [13] to hypervascular well differentiated HCC that often contain steatosis lesions [14, 15]. Steatosis leads to restricted diffusion [16, 17]; therefore, this effect may be present in this study. In the present study, minimum ADC in poorly differentiated HCC was significantly lower than both well and moderately differentiated HCC excluding steatosis-containing lesions. This result supports the concept that steatosis affects minimum ADC.

The effective parameters of ADC histograms for distinguishing histological differentiation are dependent on sites originating tumors. Woo et al reported that standard deviation and 75th,90th, and 95th percentiles were useful for differentiating low from high grade in endometrial cancer [18]. The results were reflected by the tissue necrosis in high grade endometrial cancer. On the other hand, high grade HCC usually showing hypercellularity, necrosis, or cystic change was rare. Suo et al. also reported that the mean, minimum, maximum, and 10th, 25th, 50th, 75th, and 90th percentile ADCs were significantly lower, while skewness and entropy ADCs were significantly higher in malignant lesions compared with benign ones in breast tumor [19]. The results reflected hypercellularity and pathological heterogeneity. The present study showed only minimum ADC and skewness showed no significant differences among degrees of tumor differentiation. We supposed these results reflected hypercellularity of HCC and relative pathological homogeneity compared with breast tumors. The pathological homogeneity may lead to making the distinction between tumor differentiation classifications difficult. Therefore, effective parameters of ADC histograms in predicting tumor histological grade were dependent on the characteristics of the tumor. The usefulness of kurtosis was less reported in previous studies of ADC histograms in abdominal and pelvic tumors except for a few reports [18–21], and the present study also showed no significant differences according to tumor differentiation.

The *b*-values might have affected the results of 3D analysis of ADC histograms. Nasu et al reported the mean ADC values of well, moderately, and poorly differentiated HCC were much higher than those of the present study [6]. They used *b*-values of 0 and 500 s/mm^2^, and used lower *b*-values than we used. On the other hand, Heo et al reported similar results to the present study, although mean ADC values of poorly differentiated HCC were significantly lower than those of well and moderately differentiated HCC [9]. They used *b*-values of 0 and 1000 s/mm^2^. Therefore, the standardization of *b*-values is important.

There are several limitations in the present study. First, the sample size of tumor histological differentiation was biased due to many moderately differentiated HCCs. The reason was that operation candidates were collected in the study. Second, some fusion errors from different slice thicknesses might affect the result. This is an important issue for improving this method. Third, we excluded the tumors located in the lateral segment because of cardiac motion. Cardiac motion causes negligible artifact (signal loss). This is a disadvantage of DWI. Recently, some methods have been proposed to reduce the cardiac motion [22–24]. These methods should be tried in the future when performing clinical routines.

## Conclusions

In conclusion, minimum ADC was the most promising parameter for distinguishing poorly differentiated HCC from the other histological grades on 3D analysis of ADC histograms.

## Abbreviations

3D-VIBE: Three-dimensional volumetric interpolated breath-hold examination
ADC: Apparent diffusion coefficient
CHESS: Chemical shift selective
DWI: Diffusion-weighted imaging
FOV: Field of view
Gd-EOB-DTPA: Gadolinium-ethoxybenzyl-diethylenetriamine pentaacetic acid
HCC: Hepatocellular carcinoma
ROI: Region of interest
T1WI: T1-weighted imaging
T2WI: T2-weighted imaging
TR/TE: Repetition time/Echo time
